# Latitudinal cline of chronotype

**DOI:** 10.1038/s41598-017-05797-w

**Published:** 2017-07-14

**Authors:** Mario André Leocadio-Miguel, Fernando Mazzili Louzada, Leandro Lourenção Duarte, Roberta Peixoto Areas, Marilene Alam, Marcelo Ventura Freire, John Fontenele-Araujo, Luiz Menna-Barreto, Mario Pedrazzoli

**Affiliations:** 1 Universidade Federal do Rio Grande do Norte, Departamento de Fisiologia, Natal, Brazil; 2 Universidade Federal do Paraná, Departamento de Fisiologia, Curitiba, Brazil; 3 Universidade Federal do Recôncavo da Bahia, Centro de Ciência da Saúde, Santo Antônio de Jesus, Brazil; 4 Universidade de São Paulo, Escola de Artes, Ciências e Humanidades, São Paulo, Brazil; 5 Universidade Federal de Pelotas, Departamento de Farmacologia e Fisiologia, Pelotas, Brazil

## Abstract

The rotation of the Earth around its own axis and around the sun determines the characteristics of the light/dark cycle, the most stable and ancient 24 h temporal cue for all organisms. Due to the tilt in the earth’s axis in relation to the plane of the earth’s orbit around the sun, sunlight reaches the Earth differentially depending on the latitude. The timing of circadian rhythms varies among individuals of a given population and biological and environmental factors underlie this variability. In the present study, we tested the hypothesis that latitude is associated to the regulation of circadian rhythm in humans. We have studied chronotype profiles across latitudinal cline from around 0° to 32° South in Brazil in a sample of 12,884 volunteers living in the same time zone. The analysis of the results revealed that humans are sensitive to the different sunlight signals tied to differences in latitude, resulting in a morning to evening latitudinal cline of chronotypes towards higher latitudes.

## Introduction

The concept of chronotype, which is the expression of diurnal preferences or circadian phenotype, including rise and bedtime preferences, has received a strong consideration from studies in human chronobiology^1^. It is generally accepted that the chronotype distribution in populations is the same, no matter where the population is geographically localized. Additionally, independent studies in different locations around the world use the same cut-off points for chronotype classification as originally proposed by Horne and Ostberg^2^, regardless that daily rhythmicity is determined by the interaction between the signal generated by the endogenous circadian pacemaker and the environmental synchronizer agents. While the circadian pacemaker provides a free-running period of approximately 24 h, external synchronizing agents modulate both the period and phase of the endogenous rhythms to match the environmental signal^3, 4^.

The light/dark cycle generated by the rotation of earth around its own axis and around the sun has been considered the strongest zeitgeber that entrains circadian rhythms^5^. However, artificial light, social interaction, physical exercise and feeding patterns have also been implicated as important time cues, indicating that multiple zeitgebers interact to synchronize the circadian rhythms of different species, including humans^6^. Thus, for instance, depending on the strength of zeitgebers and on the length of an individual’s free-running period, the phase of entrainment may be different, giving rise to a broad distribution of wake and sleep times^7^. Additionally, It has been shown that the light/dark cycle has a stronger effect on synchronizing circadian rhythms, when compared to social timing^8^. This finding is supported by studies with blind people, particularly those with enucleated eyes, who live in a desynchronized state the non-24 h sleep/wake disorder, despite the large number of additional potential zeitgebers they are subjected every day^9^.

Real-life human epidemiological experiments offer the ideal scenario to understand how the composition of zeitgebers works and to unveil the role of the light/dark cycle as a relevant synchronizing signal for humans, as long as controlled experiments in sleep labs can primarily provide limited heuristic information that should be subjected to confirmation in real life. Therefore, the circadian system evolved as a set of mechanisms sensitive to the perception and response to natural conditions that have been marked by the robust and stable solar light emanating throughout the evolutionary processes.

The Brazilian territory is situated along a range of latitudes from the equator line to approximately 33 degrees south and offers an excellent opportunity to test possible impacts of latitude on circadian parameters. Considering this geographical advantage, Morningness-eveningness preferences have been used for many years as an approximation of the endogenous circadian phase, and they are easily measured by the Horne-Ostberg (HO) questionnaire. In the present study, we administered an on-line version of this questionnaire to a large sample of the population along all latitudinal clines of the Brazilian territory to test the hypothesis that latitude is associated to the regulation of circadian rhythm in humans.

## Results

### Chronotype along the Latitudinal Cline

**Figure 1:**
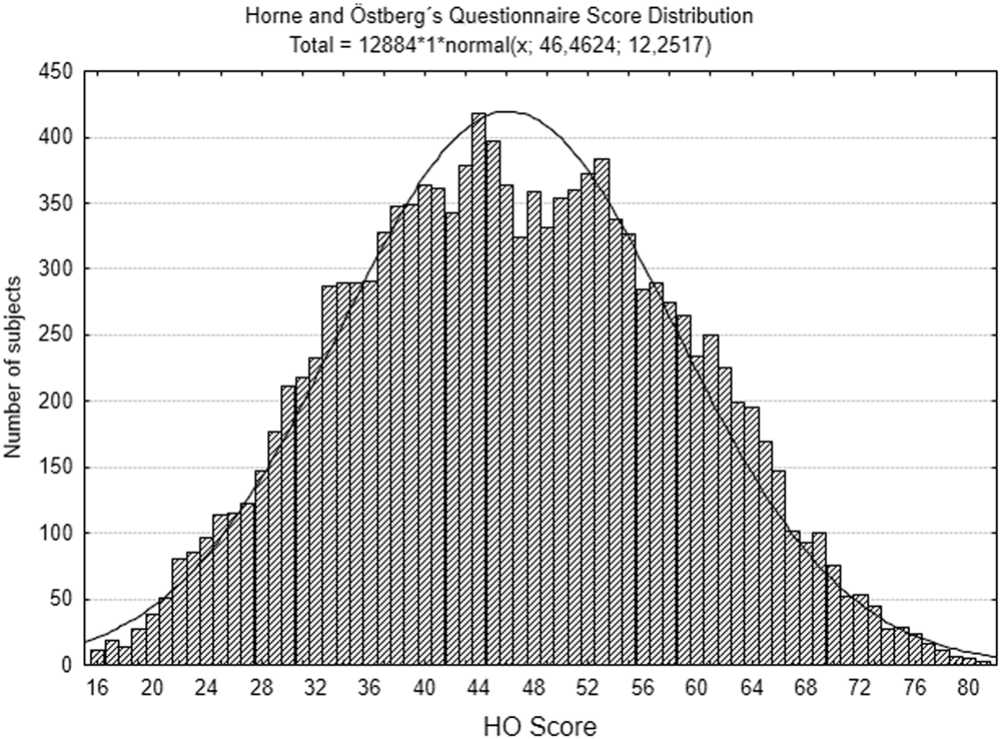
Distribution of HO scores for the entire sample of data. Each bar for each score represents the frequency (number of subjects). Low scores represent evening types and high scores morning types.

HO answers were found reliable according to Cronbach’s test, indicating internal consistency (α = 0.860). The mean HO score was 46.4 (ranging from 16 to 81). However, the distribution of HO scores within the sample was not normal according to Kolmogorov-Smirnov test (p < 0.0001). The descriptive analysis of the distribution reveals that median is 46, lower and upper quartiles are 37 and 55 respectively, Skewness is 0.050 (SE 0.021), Kurtosis – 0.56 (SE 0.04). The curve distribution of the entire sample is shown in Fig. 1. Descriptive analysis and graphics for each range of latitude may be seen as Supplementary material S-I.

We conducted a first regression of HO scores only on the covariates (age, longitude, and solar irradiation when the subjects filled the online questionnaire) and cofactors (sex, daylight saving time (DST), and season) (R2 = 0.05964, F(8,12845) = 101.8, p < 2.2e-16) and a second regression including the annual average solar irradiation, sunrise time, sunset time and daylight duration in March equinox, June, and December solstices for each volunteer (R2 = 0.06667, F(27,12853) = 33.8, p < 2.2e-16). Testing for nested models, we obtained a significant reduction of residual sum of squares (F(2,12884) = 31.983, p = 1.395e-14), which means that annual average solar irradiation exerts systematic effect on HO score even after controlling for age, longitude, sex, DST, season, and solar irradiation when the subjects filled the online questionnaire. We also obtained an effect size estimate of Cohen’s f2 = (0.06352-0.05964)/(1-0.06352) = 0.004143174. We tested the residuals of the second regression with Kolmogorov-Smirnov normality test and obtained a significant result (D = 0.42598, p < 2.2e-16).

**Figure 2:**
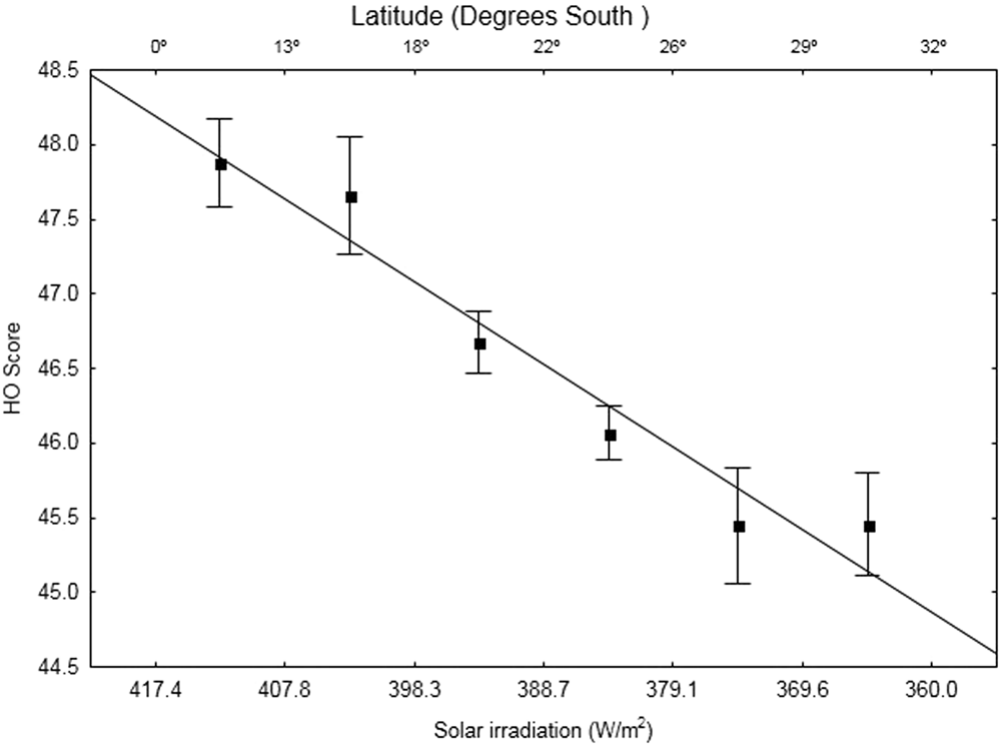
Mean HO scores (±SE) along a latitudinal cline, showing corresponding annual average of solar irradiation level (W/m^2^).

Furthermore, after we have categorized latitudinal ranges based on solar irradiation levels (6 bins of approximately 10 W/m^2^), we found that it was maintained the significant association between latitude and HO score, even when corrected by longitude (Quade^0^ s rank analysis of covariance, F(5,12784) = 7.22, p = 9.20e-07), reinforcing the clear tendency towards eveningness in higher latitudes or lower mean irradiation levels (Fig. 2).

**Figure 3:**
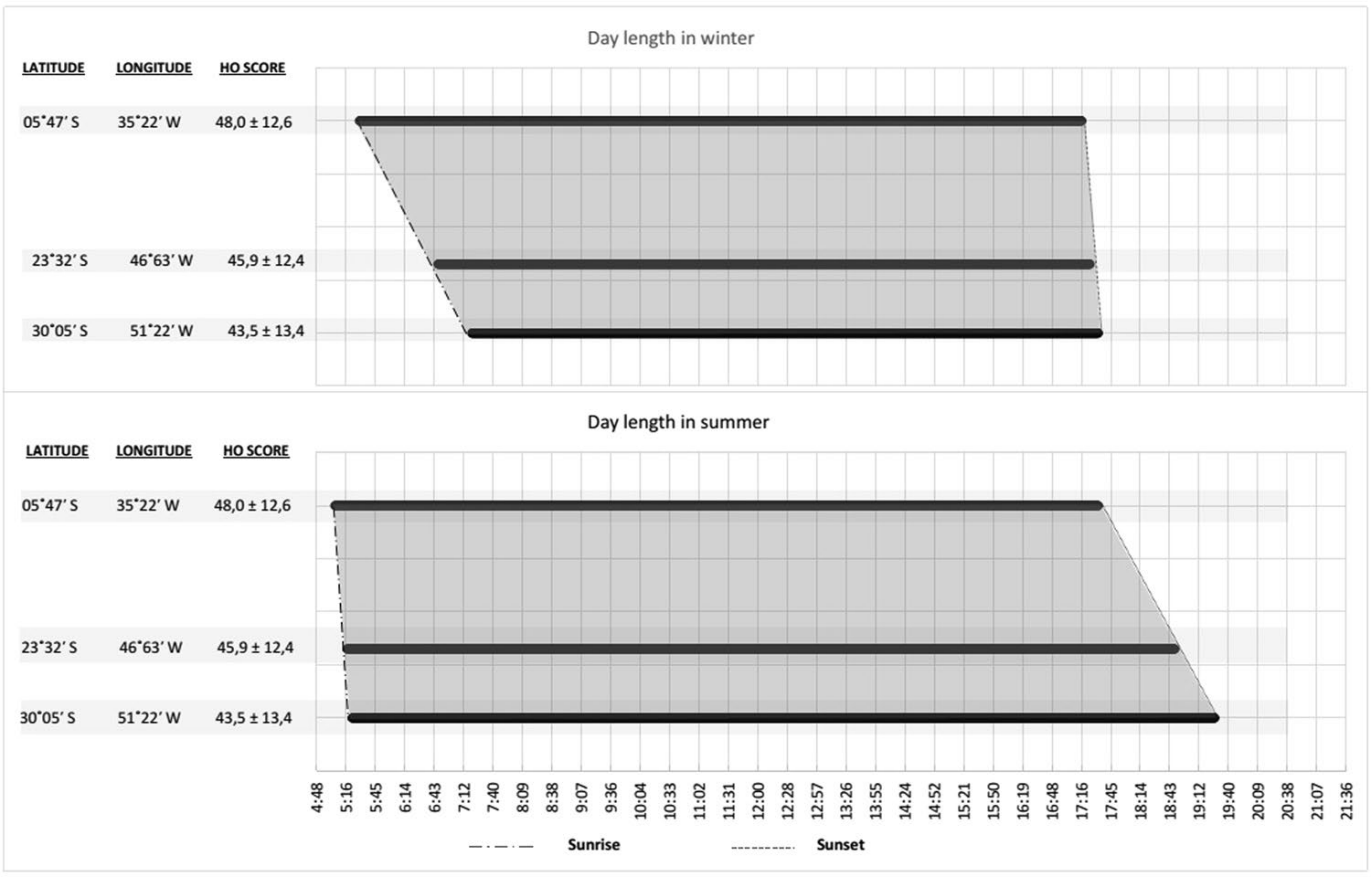
Sunrise, sunset and day length in the three cities depicted in summer and winter solstices. The grey area represents day length. During the summer, the increase in day length at higher latitudes is associated primarily with a delay of sunset (compared with low latitudes). During the winter, the decrease in day length at higher latitudes is associated primarily with a delay of sunrise.

To further characterize the chronotype latitudinal cline, we chose three representative cities along the Brazilian coast, based on the following criteria: population size, geographical position along the coast (where 92 percent of the Brazilian population lives) and number of volunteers. These cities represent the general pattern of occupation in Brazil, characterized by a westward-southward progression from the northeast towards the south of Brazil (Supplementary material S-II). Then, we performed a comparison of the chronotype and day length data from three large cities included in the study: Natal (5°46′ south, 35°12′ west), São Paulo (23°33′ south, 46°37′ west) and Porto Alegre (30°2′ south, 51°13′ West). The populations of these cities are at least eight hundred thousand people, and these cities are located at three different latitudes and longitudes, thus following the geography of the Brazilian coast and representing, at a small enough scale, the general exposition to light/dark cycle of 92% of Brazilian population. This set of data allowed us to observe sunrise and sunset relative to the averaged HO scores and to verify that higher HO scores at low latitudes match with early sunrise in the winter and with early sunset in the summer (Fig. 3).

## Discussion

Previous studies have shown distinct distributions of morningness-eveningness in different populations^10–13^, linking geographi- cal characteristics to chronotype expression, such as longitude^14, 15^, and latitude^16^, including some of our own experiments that corroborate the existence of a latitudinal cline in the chronotype, in both the behavioural plan^13^, and in the molecular plan (submitted). To our knowledge, however, this study is the first to be performed in a single country within the same time zone and comprising a wide range of latitudes, with a large sample size sharing strong cultural traits and the same spoken language. In accordance with our hypothesis, we have shown findings that indicate that the farther away the population is from the equator line, the more significant is the shift of the chronotype distribution towards later chronotypes. To explain our results, multiple theoretical backgrounds of circadian synchronization mechanisms must be considered.

To start this discussion, it must be clear that although latitudinal parallels can be considered as a human construct, they reflect physical features of geography. Each latitude represents a set of complex environmental variables, including temperature oscillation, solar irradiation, and others abiotic factors. Consequently, latitudinal specific daily and annual variations of factors, such as solar irradiation and temperature, give rise to evolutionary pressures influencing the phenotypic expression of living beings.

Additionally, it should be emphasized to the reader that when we observe the patterns of light/dark along the Brazilian coast (where the majority of Brazilian population lives), it is possible to realize that the dawn and dusk are shaped as an angular line with around 15 degrees of longitude from northeast to south, which is approximately parallel to the coast. This arrangement results, for part of the year, in sunrise (in the summer) or sunset (in the winter) happening almost at the same time in cities located in northeast and south of Brazil, even though they are at distinct longitudes (Fig. 3), and Supplementary material (S2, S3 and S4).

Undoubtedly, discrete transition signals, such as dusk and dawn, result in non-parametric effects of light on the circadian rhythms of humans. Taken into account the human phase response curve to light^17, 18^, the lengthening of the light phase of day during the summer, as the latitude increases, makes light exposure to occur in a phase sensitive to circadian rhythm delays, comparing southeast to the northeast of Brazil. Again, during the winter, the shortening in the day length, as the latitude increases, is primarily associated with a delay in the time of sunrise and with low levels of solar irradiance (see Fig. 3), reducing the exposure to light during a phase sensitive to phase advances, for people living in the south. In summary, the phase differences related to sunlight exposure between higher and lower latitudes, in Brazil, act in the same direction in the summer (earlier sunrises at low latitudes) and winter (late sunsets at high latitudes), resulting in a delay of circadian rhythms at higher latitudes. Therefore, these results indicate that populations living at higher-latitude regions have a tendency towards eveningness.

Moreover, our chronotype is not supposed to be only subjected to phasic, non-parametric synchronizing effects of light, but also to tonic, or parametric influence of light. Irrespectively of light/dark transitions, daily and annual modulation of solar irradiation depends on latitude, leading to a possibly distinct latitude-specific selection pressure^19^. Thus, a possible explanation for the increased tendency towards eveningness farther from the equator is that as the mean solar irradiation levels decreases, the average strength of the zeitgeber is reduced, which would lead to a higher probability of weakening the circadian entrainment. This is based on the fact that, in experimental conditions with low intensity light, people usually express an endogenous circadian period that is longer than 24 h^20–22^ and the net result is an increased tendency towards eveningness. As a complementary interpretation, also linked to tonic influence of light, the progressive decrease in mean solar irradiation levels, as latitude increases, would change the coupling between the light-entrainable circadian oscillators, leading to a higher degree of coupling and a reduction in their velocity, which would culminate in longer periods and a delayed-phase pattern, when compared to low latitude conditions^23^.

Accordingly, to determine the relationship between latitude and the synchronization of circadian rhythms, it was proposed that the amplitude of the theoretical phase response curve may be reduced as one goes from the equator to either North or South^24^. This fact would help to explain higher HO scores (morningness) under conditions of elevated mean solar irradiation, as well as lower HO scores (eveningness) under decreased mean solar irradiation. Recently, it has been reported that reduced exposure to natural sunlight from the light-dark cycle and the increased use of artificial electrical light can alter the human circadian physiology. Using only sunlight while living outdoors diminishes the individual differences in circadian timing and leads to an earlier sleep phase^25^; this is in accordance with the entrainment theory for circadian clocks in the presence of strong zeitgebers^8^. Acknowledging that it would only be possible to infer about the association between outdoor living and chronotype by measuring individual patterns of light exposition, the results from Wright *et al*.^25^ seem to corroborate our findings for lower latitudes in Brazil, were the strength of the zeitgeber is presumed to be stronger. Obviously, there is a need for further studies designed to specifically address individual light exposition in addition to the variables we indeed studied here.

Thus, our main result - increased prevalence of eveningness at higher latitudes - can be better clarified by a multivariable functional model that considers the diversity of physiological mechanisms of temporal adjustments. Our belief is that the different chronotype distributions for lower and higher latitudes are explained by both phasic and tonic responses to light. In fact, timing (phase), the pattern of the light-dark transitions and the strength of the zeitgeber should act on the circadian system to produce circadian behaviours, which can be predicted from the properties of the circadian system^26, 27^. The integration of the light/dark temporal and intensity signals with a subject’s internal timing may result in the phenotype of phase entrainment observed along the latitudinal cline in Brazil. This assumption leads us to propose that the chronotype is a mutable intrinsic property and not a fixed characteristic of the human circadian system; instead, it should be, at least in part, a flexible outcome resulting from the light/dark signaling that acts on an individual’s internal timing, which is regulated by genetic and ontogenetic factors^28^. Furthermore, our results are in line with the previously proposed environment hypothesis^16, 29^, since we consider as tied to latitude both temperature (that increased towards equator and leads to morningness^30^ and amplitude of sunset and sunrise (increases towards the poles and leads to eveningness^10^.

Social and cultural factors cannot be ruled out. In fact, it is likely, that both light (artificial and natural) and social/cultural patterns interact to compose an environment with multiple zeitgebers and it is the basis for the understanding of chronotype. For instance, the comparison of sleep patterns between rural and urban populations reinforces the role of the exposure to light, related to distinct social or cultural organizations, in determining the sleep phenotype tied to chronotype^31–35^. Therefore, we consider that the prevalence of morning-oriented people in northeast Brazil could be justified not only by the effects of the variation in sunrise, sunset and light intensity, but also by cultural, or social factors that modulate an individual’s exposure to the natural light/dark cycle, which should contribute to the expression of the circadian timekeeping system.

In the contemporary society, we tend to perceive time as homogeneous and globalized, which sets aside the local temporality, represented here as the latitudinal cline. In addition, this misperception of time brings to our mind, stealthily, the idea of the flatness of the Earth^36^. This contemporary timing ideology and the actions that follow from it generate temporal tensions in humans, such as the so-called social jet lag^37^. Indeed, this temporal tension is a characteristic of contemporary urban society and is a result of historically established temporal social schedules (clocks), of the use of artificial light and indoor living, which act as stimuli on the human biological timing systems. This sense of local temporality conflicts with the culturally imposed globalized sense of daily time, which may have deleterious consequences to human health. As a conclusion, we have confirmed that the differential variation in the natural light/dark cycle, driven by latitude, may be crucial for the entrainment process of the human circadian system.

## Methods

### Measurement Instrument

To determine their chronotype, we asked subjects to complete a web-based platform of the Brazilian Portuguese version of the Horne and Östberg’s Morningness-Eveningness Questionnaire^38^ (available at http://www.each.usp.br/crono). The answers from the volunteers were kept in a structured database. The volunteers were also asked to provide demographic information about their gender, city of residence, age and work/study routines on the same webpage.

### Geographic Parameters

The Brazilian territory is situated along a latitude range from 05°16′ north to 33°45′ south and a longitude range from 34°47′ to 73°59′ west. Most of the country’s population (92% of approximately 194 million people) live in the same time zone, concentrated at or near the east coast. In our study, we analyzed subjects living in the latitude range from 0° to 32°33′ and the longitude range from 34°50′ to 57°05′, comprising the time zone UTC/GMT – 3.

The latitude and longitude of the cities included in the study were obtained from the website http://www.satsig.net/maps/lat- long-finder.htm. The irradiation (W/m^2^) data by latitude was acquired from the NASA website (http://aom.giss.nasa.gov). We retrieved monthly average data and calculated the annual mean for each latitude degree.

### Sample

A group of 12,884 volunteers living in the same time zone were chosen from the database. Exclusion criteria included subjects younger than 18 years old, subjects who lived outside the Brazilian east coast time zone and shiftworkers. Our sample was relatively young: 58.6% of the selected subjects were 30 years old or younger. The mean age was 31.3 ± 10.5 years, ranging from 18 to 75 years, and 69.9% were females.

This study conformed to international ethical standards based on the Declaration of Helsinki. The local Committee on Ethics of the University of São Paulo approved this study. Informed consent was obtained from each subject.

### Statistical Analysis

We used Cronbach’s α to test the reliability of the HO answers throughout the sample. We utilized the Kolmogorov-Smirnov test to verify that the HO scores adhered to a normal distribution. Quade’s rank analysis of covariance was performed.

We conducted a sequential multiple regression of HO scores on annual average solar irradiation, controlling for the covariates age, longitude, and solar irradiation when the subjects filled the online questionnaire, sunrise time, sunset time and sunlight duration in March equinox, June, and December solstices for each volunteer and for the cofactors sex, season of the year on the moment of the filling of the questionnaire and whether there was daylight saving time (DST) on the region of the subject. We used the statistical software R version 3.3.1. and Statsoft Statistica v. 10^TM^ for all analyses.

## Electronic supplementary material

Supplementary Material
