# Supplementary material for: Latitudinal cline of chronotype
Source: Sci Rep. 2017 Jul 14;7:5437. doi: 10.1038/s41598-017-05797-w (PMC5511182; doi:10.1038/s41598-017-05797-w)
Supplement: Supplementary file 1 — Supplementary Material [file 41598_2017_5797_MOESM1_ESM.pdf]

Latitudinal cline of chronotype

Mario André Leocadio Miguel, Fernando Mazzili Louzada, Leandro Lourenção Duarte, Roberta Peixoto Areas, Marilene Alam, Marcelo Ventura Freire, John Fontenele Araujo, Luiz Menna-Barreto, Mario Pedrazzoli

Supplementary Online Material

Supplementary Material: S-I

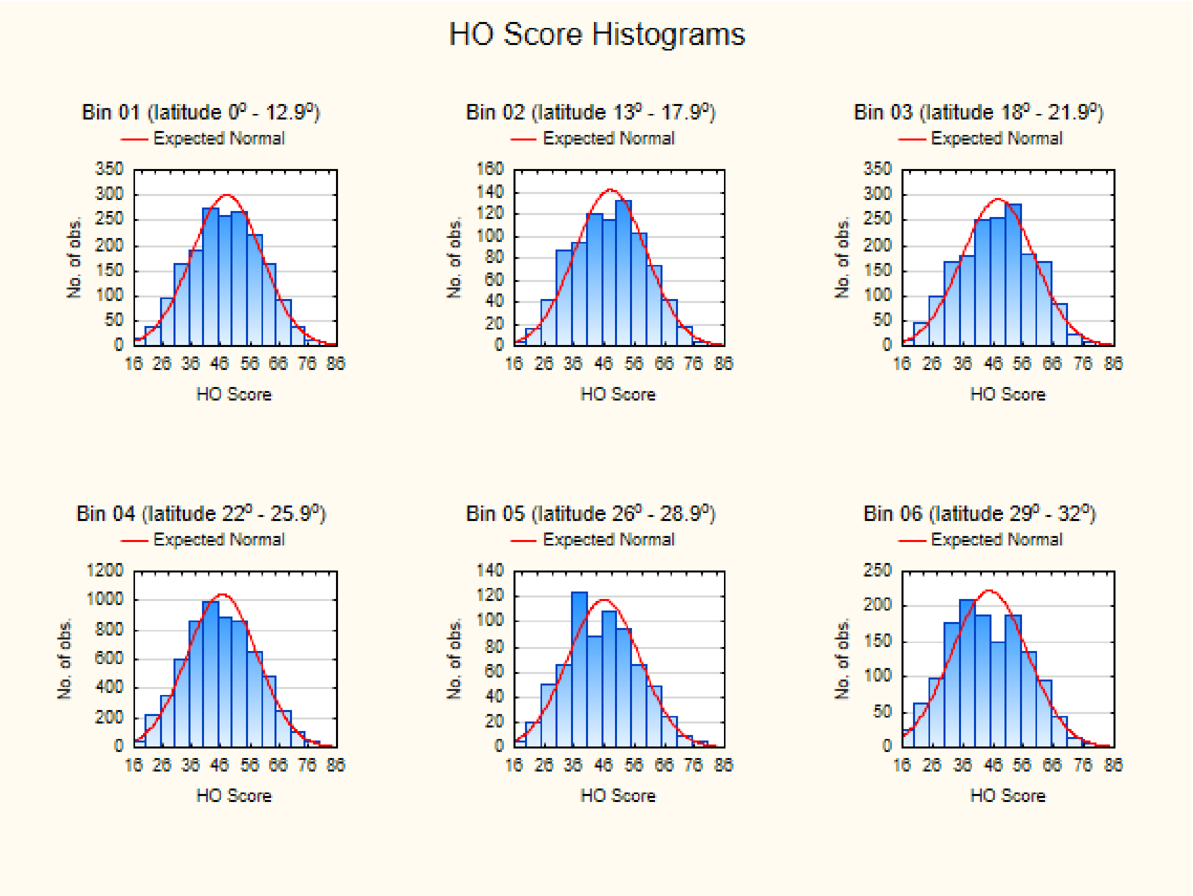

Figure S1. HO Score histograms, according to latitude bins.

Table S1. Descriptive analysis for HO score histograms, according to latitude bins.

	VALID N	MEAN	MEDIAN	SKEWNESS	SKEWNESS (S.E.)	KURTOSIS	KURTOSIS (S.E.)
**BIN 1**	1830	47.99	48.00	-0.039	0.057	-0.5089	0.1143
**BIN 2**	846	48.00	48.00	-0.001	0.084	-0.6032	0.1679
**BIN 3**	1754	47.63	48.00	-0.075	0.058	-0.6054	0.1168
**BIN 4**	6347	46.41	46.00	0.079	0.030	-0.4929	0.0614
**BIN 5**	707	45.79	46.00	0.154	0.092	-0.4863	0.1835
**BIN 6**	1399	44.76	44.00	0.116	0.065	-0.6378	0.1307

Supplementary material: S-II

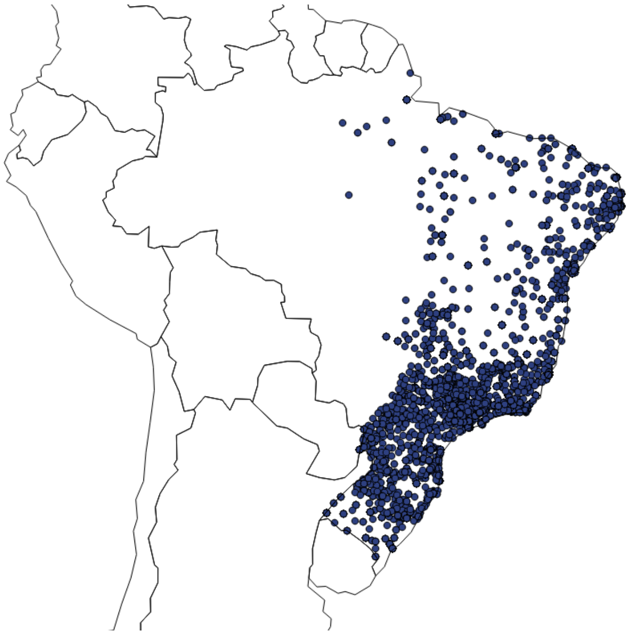

Figure S2. Distribution of HO respondents, according to the Brazilian map at time zone -3 UTC. Each blue dot represents a city (latitude and longitude) from where we collected data. Image generated by the authors through the geoprocessing Software QGIS, v. 2.18.3. http://www.qgis.org.

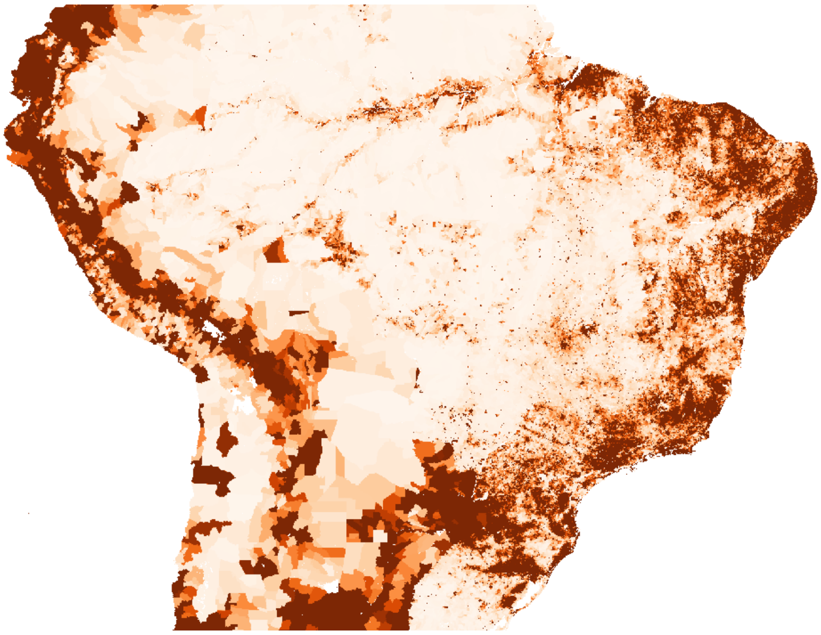

Figure S3. Distribution and demographic concentration of Brazilian population. Dark color indicates higher population density. Image generated by the authors through the geoprocessing Software QGIS, v. 2.18.3. *http://www.qgis.org*. Data set available (under C.C. 4.0 license) from Center for International Earth Science Information Network - CIESIN - Columbia University. 2016. Gridded Population of the World, Version 4 (GPWv4): Population Density. Palisades, NY: NASA Socioeconomic Data and Applications Center (SEDAC). Available at http://sedac.ciesin.columbia.edu/data/set/gpw-v4-population-density.

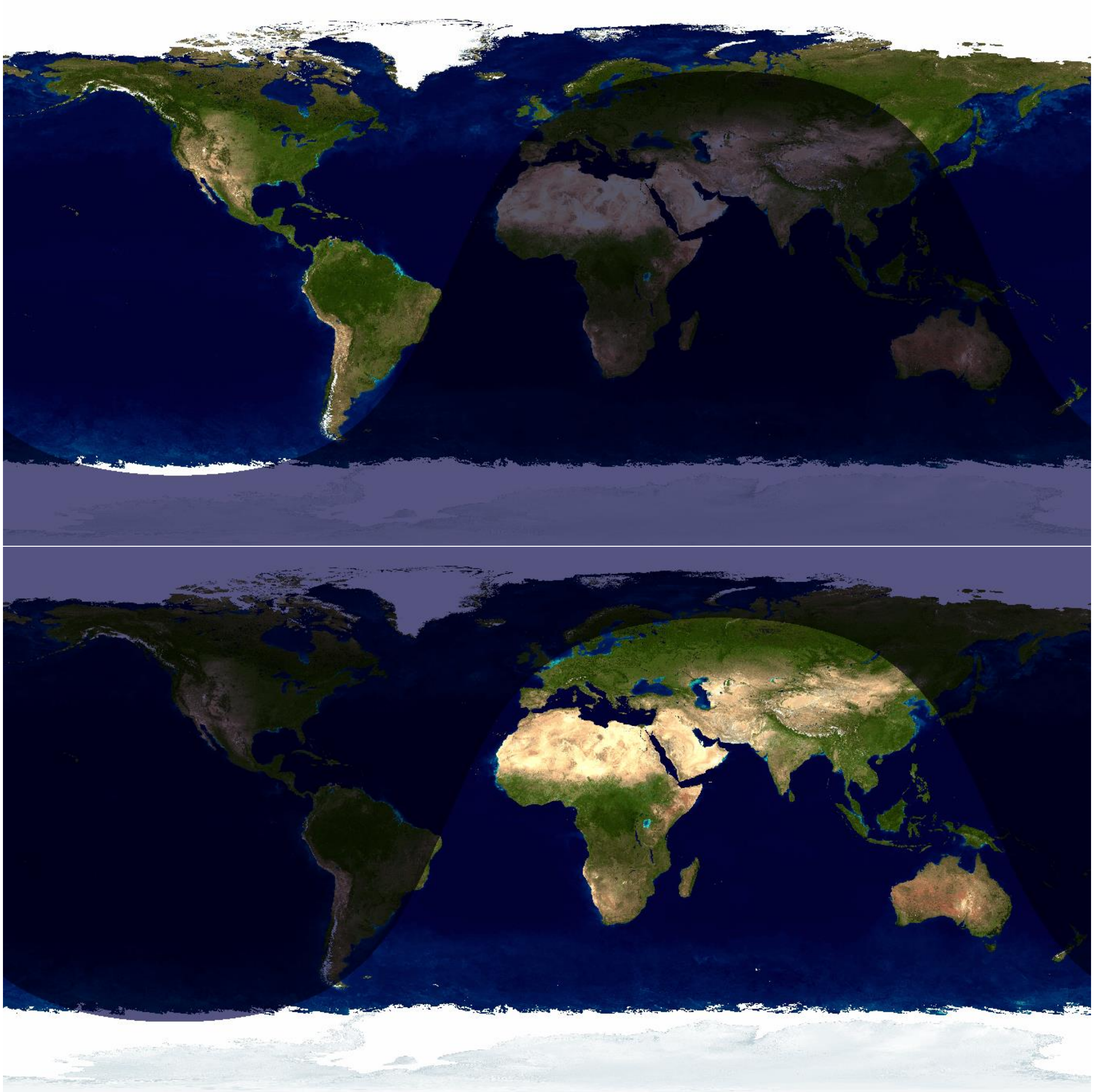

Sunset along Brazilian coast in June 21th - 5:15 PM (upper panel). Sunrise along Brazilian coast in December, 21th - 5:30 AM (lower panel). Images created by the authors, through the use of the software Home Planet for Windows (Release3.3a – Freeware – by John Walker, available at http://www.fourmilab.ch). The images show similar dusk and dawn times in cities along the Brazilian coast although they are localized at different longitudes.

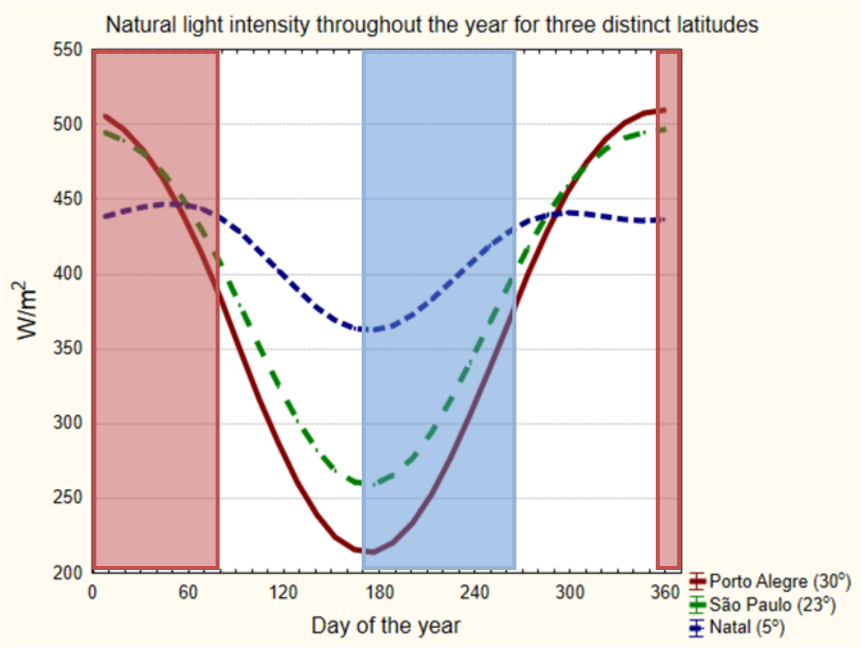

Figure S5. Natural light intensity throughout the year for three distinct latitudes from North (5º) towards South (30º): Porto Alegre, São Paulo and Natal. The X-axis represents days of the year around day 180 (mid-winter). The Y-axis represents natural light intensity (W/m^2). In blue winter season in red summer.
